# Recent Advances in Nanowire-Based Wearable Physical Sensors

**DOI:** 10.3390/bios13121025

**Published:** 2023-12-11

**Authors:** Junlin Gu, Yunfei Shen, Shijia Tian, Zhaoguo Xue, Xianhong Meng

**Affiliations:** National Key Laboratory of Strength and Structural Integrity, School of Aeronautic Science and Engineering, Beihang University, Beijing 100191, China

**Keywords:** nanowires, wearable electronics, wearable sensors, health monitoring, human–machine interfaces

## Abstract

Wearable electronics is a technology that closely integrates electronic devices with the human body or clothing, which can realize human–computer interaction, health monitoring, smart medical, and other functions. Wearable physical sensors are an important part of wearable electronics. They can sense various physical signals from the human body or the surrounding environment and convert them into electrical signals for processing and analysis. Nanowires (NW) have unique properties such as a high surface-to-volume ratio, high flexibility, high carrier mobility, a tunable bandgap, a large piezoresistive coefficient, and a strong light–matter interaction. They are one of the ideal candidates for the fabrication of wearable physical sensors with high sensitivity, fast response, and low power consumption. In this review, we summarize recent advances in various types of NW-based wearable physical sensors, specifically including mechanical, photoelectric, temperature, and multifunctional sensors. The discussion revolves around the structural design, sensing mechanisms, manufacture, and practical applications of these sensors, highlighting the positive role that NWs play in the sensing process. Finally, we present the conclusions with perspectives on current challenges and future opportunities in this field.

## 1. Introduction

In recent decades, wearable electronics have undergone extensive research and development in various fields, such as human health monitoring [1,2,3], human–machine interfaces [4,5,6,7], and the Internet of Things (IoT) [8,9], due to their flexible/stretchable, lightweight, and skin-friendly features [10,11,12,13,14,15,16,17,18,19,20,21,22]. Wearable physical sensors are an important part of wearable electronics and enable real-time and continuous monitoring of various physical signals of the human body and the environment. These sensors can be conformally attached to the human skin or clothing in the form of patches [23,24], tattoos [25,26], or fabrics [27,28]. By converting diverse physical signals into measurable electrical signals, these sensors facilitate sensing functions for physical quantities like pressure, strain, light, temperature, and humidity, reflecting the human body’s motion, physiological and pathological status, or the surrounding conditions.

Nanomaterials, characterized by their high specific surface area, mechanical properties, and electrical properties, have emerged as promising sensitive materials for wearable sensors [29,30,31]. Graphene [32,33], carbon nanotubes (CNTs) [34,35], nanowires (NWs) [5,36,37,38], nanoparticles [39,40], quantum dots (QDs) [41,42], nano-patterned thin films [43,44], nanostructured materials [45,46], nanocomposite materials [47,48], and more have been employed in this domain. Among them, NWs are one-dimensional nanostructures with diameters ranging from a few nanometers to hundreds of nanometers and lengths up to several micrometers or even millimeters. They have unique properties such as a high surface-to-volume ratio, high flexibility, a tunable bandgap, high carrier mobility, a large piezoresistive coefficient, and a strong light–matter interaction. These properties make NWs ideal candidates for the fabrication of wearable physical sensors with high sensitivity, fast responses, and low power consumption. In addition to sensing properties, the nano dimension of NWs allows their percolation network to have a certain degree of optical transparency [49], which has important applications in wearable electronics. The transparency is related to the thickness of the film, which makes spray-coating a good choice for preparing transparent NW networks for wearable sensors [50]. Moreover, NWs can be synthesized by various methods with different materials, such as metals, semiconductors, and oxides, which provide a rich diversity of NW-based wearable physical sensors for different applications [5,29,30,31,36].

**Figure 1:**
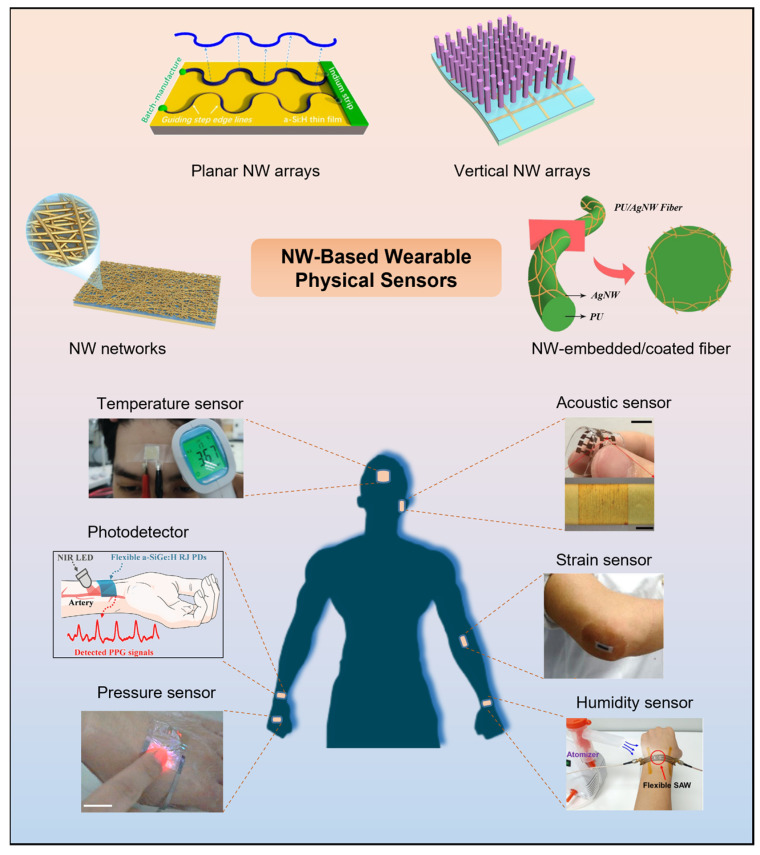
Schematic diagram of the structure modes and sensor types of NW-based sensors. The structure types of NWs can be mainly divided into networks, planar arrays, vertical arrays, and composite fibers. Reprinted with permission from [51]. Copyright 2022, American Chemical Society. Reprinted with permission from [52]. Copyright 2017, American Chemical Society. Reprinted with permission from [53]. Copyright 2019, Elsevier. Reprinted with permission from [54]. Copyright 2019, American Chemical Society. The representative types of NW-based wearable physical sensors for applications in the human body, including temperature sensors, photodetectors, pressure sensors, acoustic sensors, strain sensors, and humidity sensors: Reprinted with permission from [55]. Copyright 2022, The Royal Society of Chemistry. Reprinted with permission from [56]. Copyright 2022, Wiley-VCH. Reprinted with permission from [50]. Copyright 2018, Springer Nature. Reprinted with permission from [57]. Copyright 2020, Wiley-VCH. Reprinted with permission from [58]. Copyright 2018, Springer Nature. Reprinted with permission from [59]. Copyright 2020, American Chemical Society.

In this review, we provide a comprehensive overview of the recent advances in various types of NW-based wearable physical sensors in the past five years, encompassing mechanical, photoelectric, temperature, humidity, and multifunctional physical sensors. Figure 1 presents various wearable sensor applications using NWs with diverse materials and arrangements, such as metal, semiconductor, and oxide NWs in the form of networks, planar arrays, vertical arrays, and composite fibers. We introduce the sensing mechanism, design strategies, fabrication methods, and practical application of representative works in each section and discuss the roles of NWs in enhancing the performance, functionality, and reliability of the wearable sensors. Finally, we conclude this review and provide perspectives on current challenges and future opportunities for NW-based wearable sensors.

## 2. NW-Based Wearable Mechanical Sensors

Wearable mechanical sensors can detect and measure the mechanical signals on the wearer’s skin surface, such as strain and pressure. These signals can reflect the wearer’s body motion, gestures, vital signs, and health conditions. Due to their excellent mechanical and sensing properties, NWs have been widely used as active or passive elements in wearable mechanical sensors. In this section, we introduce the latest developments in strain and pressure sensors.

### 2.1. Strain Sensors

Strain sensors play a crucial role in the realm of wearable technology. These sensors have diverse applications in human wearables, enabling the measurement of subtle changes in human motion, gesture, and facial expression. By converting the collected information into digital signals, strain sensors facilitate analysis and practical implementation in motion detection, facial expression capture, and speech recognition [31,60].

To enable the above applications, wearable strain sensors require specific performance characteristics. These include:

1. Wide-range capability: Wearable strain sensors should be capable of monitoring large-scale strains (ε≈50%) occurring in the human body, such as the bending of knee and elbow joints. This ensures the sensor’s ability to capture and measure significant deformations accurately.
2. High sensitivity: Strain sensors need to possess high sensitivity to detect small-scale strains (ε<10%) in the human body, such as facial expressions. This sensitivity allows for precise and reliable monitoring of subtle movements and micro deformations.
3. Fast response and recovery time: A desirable characteristic of strain sensors for wearable applications is a short response and recovery time. This ensures real-time monitoring capabilities, enabling immediate feedback and data acquisition.

NW-based wearable strain sensors can be broadly categorized into resistive and capacitive types, each operating on distinct principles. Compared to conventional resistive strain sensors that rely on geometric changes and piezoresistive effects, NW-based strain sensors utilizing NWs conductive fillers and polymer substrates primarily rely on disconnections, crack propagations, and tunneling effects to perceive strain [29]. On the other hand, capacitive strain sensors operate on the principle of capacitance change resulting from geometric alterations induced by strain and rely on detecting variations in capacitance to achieve strain sensing. Table 1 presents the summary of recent NW-based strain sensors with different sensing mechanisms and materials, and compares their performance parameters and features.

The sensitivity GF of the strain sensor is defined as $GF=\left(\Delta E/E_{0}\right)/\Delta \varepsilon$ [31], where Δε represents the change in strain applied to the sensor. In the case of resistive strain sensors, ΔE corresponds to the change in resistance (ΔR), while $E_{0}$ represents the initial resistance value ($R_{0}$). On the other hand, for capacitive strain sensors, ΔE denotes the change in capacitance (ΔC) and $E_{0}$ represents the initial capacitance value ($C_{0}$).

#### 2.1.1. Resistive Strain Sensors

**Figure 2:**
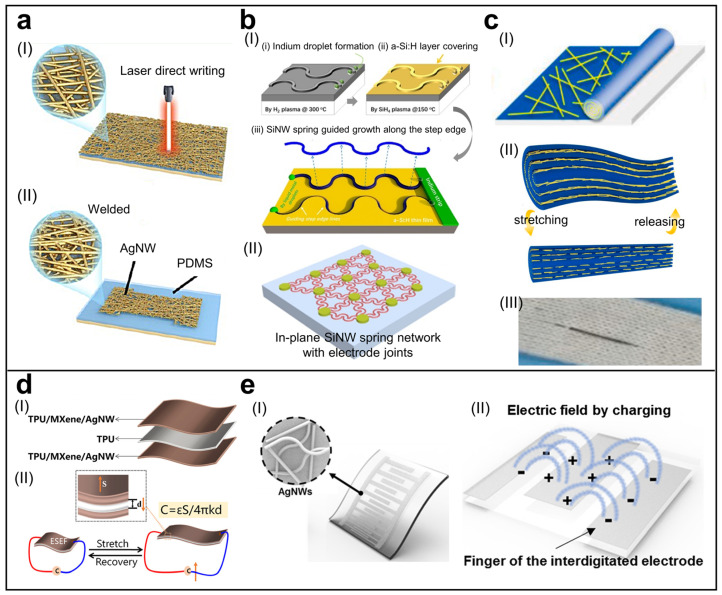
NW-based wearable strain sensors. (**a**–**c**) Resistive strain sensors. (**a**) Schematic illustration of (**I**) laser direct writing process to cut the outline of sensor and (**II**) weld AgNW network. Adapted with permission from [51]. Copyright 2022, American Chemical Society. (**b**) (**I**) SEM image and schematic illustration of an in-plane SiNW spring-guided growth along the predesigned step edge. (**II**) Schematic illustration of in-plane SiNW spring networks with electrode joints on a PDMS substrate. Adapted with permission from [52]. Copyright 2017, American Chemical Society. Adapted with permission from [83]. Copyright 2019, American Chemical Society. (**c**) (**I**) Schematic illustration of the fabrication of the fibrous strain sensor with a growth ring-like spiral structure. (**II**) Schematic for the change in AgNW networks in growth ring-like spiral structure during stretching and releasing cycle. (**III**) Optical image of knit glove integrated with the fibrous strain sensor. Adapted with permission from [64]. Copyright 2021 Elsevier Ltd. (**d**,**e**) Capacitive strain sensors. (**d**) (**I**) Exploded schematic of the electrospun sandwich-structured elastic film. (**II**) Schematic illustration of the strain-sensing mechanism of the capacitive e-skin based on electrospun sandwich-structured elastic films. Reprinted with permission from [65]. Copyright 2022 American Chemical Society. (**e**) (**I**) Schematic illustration of the interdigitated capacitive strain sensor based on AgNW. (**II**) Design Schematic of the in-plane interdigitated electrode pattern. Reprinted with permission from [49]. Copyright 2017 American Chemical Society.

In recent works, to improve the performance of NW-based resistive strain sensors, researchers have designed structures and materials to better complete wearable applications [54,55]. Sensitivity is a critical performance indicator for strain sensors in wearable applications that involve subtle strains like facial expression recognition. The connection in the conductive layer’s NWs in NW-based sensors is predominantly overlapping [61], resulting in a smaller junction area. This reduced junction area leads to decreased conductivity, ultimately diminishing the sensitivity of resistive sensors. An effective approach depicted in Figure 2a involves a welding method by reducing AgNO_3_ to Ag [51]. Through the photocatalytic reaction of AgNO_3_ under 450 nm laser irradiation, AgNWs are welded together, resulting in improved electrical conductivity and significantly enhanced sensitivity. Thermal welding is another commonly employed technique for NWs network welding. Cheng et al. fabricated an AgNWs/NFC aerogel using unidirectional freeze-casting and thermal annealing [62]. This structure combines the characteristics of an ultra-light aerogel with excellent sensitivity. In addition to welding for reducing junction resistance, the sensitivity of resistive strain sensors can also be enhanced by introducing microcracks. Yin et al. introduced microcracks into AgNWs/NFC nanopaper through pre-stretching [63], resulting in a sensor with exceptional sensitivity and an ultra-low detection limit of 0.2%.

**Table 1:** Summary of NW-based wearable strain sensors with their parameters and features.

Authors	Sensing Mechanism	Materials	Maximum Sensitivity	Stretchability	Durability	Features
Zhang et al. [51]	Resistive	AgNWs/PDMS	623.2	35%	>2000	High sensitivity
Kong et al. [64]	Resistive	AgNWs/PDMS	3	100%	>6000	Fibrous sensor, High stretchability
Du et al. [65]	Capacitive	AgNWs/Mxene/TPU	1.21	270%	>2000	Prepared by a whole electrospinning procedure
Kim et al. [49]	Capacitive	AgNWs/PDMS	1.57	100%	>1000	Transparent

Fabricating or assembling planar NW arrays on a large scale compatible with planar device fabrication and integration is crucial for developing novel NW-based electronic devices, and many pioneering efforts have been devoted to this goal. For example, some direct fabrication methods include using electron beam lithography (EBL) or nano-imprint lithography (NIL) to pattern NWs and then dry or wet etching [66,67,68,69], using epitaxial growth techniques and the vapor–liquid–solid (VLS) mechanism to grow in-plane NW with controlled orientations [70,71,72]. Furthermore, some assembly strategies include using solution flow [73], shear force guidance [74], electric/magnetic field guidance [75,76], etc., to assemble NWs into planar uniform arrays [77]. There is also a two-step anodization process for the fabrication of highly ordered nanoporous anodic aluminum oxide (AAO) templates [78], which is widely used to fabricate 1D and 2D nanostructure materials. An in-plane solid–liquid–solid (IPSLS) growth mode was proposed, where metal droplets are used to absorb amorphous silicon (a-Si:H) thin film and grow in-plane crystalline SiNWs [79,80,81,82]. More importantly, the catalyst droplets can be attracted by simple step edges to guide and grow SiNWs along the edges. This technique is compatible with the mature large-area thin-film technology. To further develop this technique, a new approach was proposed for the deterministic line-shape programming of silicon nanowires (SiNWs) for stretchable electronics through the use of catalyst droplets to grow in-plane SiNWs into arbitrary 2D patterns, as well as the reliable growth of c-SiNWs over turning tracks with different local curvatures (Figure 2(bI)) [52]. This technology provides a reliable and scalable method for the line-shape programming of SiNWs and opens up new possibilities for the creation of high-performance, bio-friendly, and stretchable electronics. Furthermore, built on this capability, the growth and monolithic integration of silicon nanowire (SiNW) springs into highly stretchable, transparent, and quasi-continuous functional networks was achieved, with a nearly perfect interconnection among the discrete electrode joints (Figure 2(bII)) [83]. These SiNW networks can be transferred onto various flexible substrates and exhibit high stretchability, transparency, and semiconducting properties. Based on the piezoresistive effect of SiNWs, stretchable and bendable strain sensors were demonstrated, showing their potential as a basis for soft electronics with high-performance SiNW.

Textile-based wearable electronics have garnered increasing attention due to their exceptional skin compatibility and conformability. These wearable electronics are typically intrinsically highly stretchable and breathable [61]. In recent studies, researchers have integrated NWs with fabrics using various methods to fabricate textile-based wearable electronics, broadly categorized into two types: yarn-integrated [61,84] and non-yarn-integrated [85,86]. Yarn-integrated fabric wearable electronics employ techniques such as electrospinning and electrospraying [84] or coating [61] to integrate NWs into yarn, fabricating textile-based wearable electronics. On the other hand, non-yarn-integrated fabric wearable electronics directly integrate NWs onto pre-fabricated fabrics using methods like heat press lamination [87], coating [86], or impregnating [85].

Building on these fabrication technologies, researchers have developed various methods to fabricate NW-based fibrous sensors [54,64]. Figure 2c demonstrates a method for fabricating fiber strain sensors using the coating plus rolling-up technique [64]. In this approach, AgNWs are coated onto a PDMS film and then rolled up to form fiber sensors with a growth ring-like spiral structure. Figure 2(cII) presents a schematic for the change in AgNW networks in the growth ring-like spiral structure during the stretching and releasing cycle. When stretched, adjacent AgNWs slid and separated, causing partial disconnection of the networks. As the strain increased, so did the disconnected regions. Because the AgNW layer was wrapped between elastic PDMS layers in the ring-like spiral structure, the networks disconnected gradually, resulting in excellent linearity. Moreover, due to the encapsulation effect and negligible viscoelasticity of PDMS, the networks changed quickly when strained and almost returned to their original state when released, leading to low hysteresis and high durability of the fiber strain sensor. 

#### 2.1.2. Capacitive Strain Sensors

NW-based capacitive strain sensors have emerged as promising devices for strain-sensing applications [49,65,87,88,89,90]. These sensors utilize the unique properties of NWs to achieve highly sensitive and accurate measurements of strain. The working principle of these sensors is based on the change in capacitance resulting from the deformation of the sensors under strain. When the sensors experience mechanical stress, their dimensions and electrical properties such as the distance between electrode plates change, leading to a variation in capacitance.

As a well-established fabrication technique, electrospinning enables the convenient integration of NWs into the electrode plates or dielectric layers of capacitive sensors. Figure 2d illustrates a flexible capacitive strain sensor that uses a TPU and AgNW-bridged Mxene nanocomposite layer as the electrode plate [65]. Benefiting from the excellent conductivity and mechanical properties of AgNW-bridged Mxene, this sensor exhibits not only outstanding sensitivity but also the ability to withstand significant deformations.

Compared to conventional capacitance sensors based on parallel electrode plates, interdigitated capacitive sensors have advantages in terms of thickness, sensitivity, linearity, and more [89]. Figure 2e presents a patterned interdigitated capacitive strain sensor made by the capillary force lithography method for AgNW [49], which not only exhibits high sensitivity and reduced thickness but also demonstrates extremely low-pressure sensitivity, effectively addressing the problem of strain-pressure cross-talk.

### 2.2. Pressure Sensors

Pressure sensors play a crucial role in the field of wearable technology, offering wide-ranging applications and holding a unique position. These devices are designed to measure the force applied to an object and convert it into an electrical signal, enabling monitoring and analysis of the object’s force state. Wearable pressure sensors facilitate various applications, including human motion detection, pulse measurement, and voice recognition [91,92,93].

Wearable pressure sensors can be categorized into four main types, each with its unique principle of operation. These categories include:

1. Resistive pressure sensors: These sensors convert pressure input into changes in resistance. By measuring the variations in electrical resistance, they indicate the applied pressure.
2. Capacitive pressure sensors: Capacitive sensors transform pressure input into changes in capacitance. They detect alterations in the electrical charge stored in a capacitor, allowing for pressure measurement.
3. Piezoelectric pressure sensors: Piezoelectric sensors generate a voltage output in response to applied pressure. They utilize the piezoelectric effect, where certain materials generate an electric charge when subjected to mechanical stress.
4. Triboelectric pressure sensors: Triboelectric sensors use the triboelectric effect to convert pressure input into electrical signals. This effect involves the generation of an electric charge through the contact and separation of two different materials.

The sensitivity S of the pressure sensor is defined as $S=\left(\Delta E/E_{0}\right)/\Delta P$ [91], where ΔP is the change in pressure applied on the sensor. For resistive, capacitive, piezoelectric, and triboelectric sensors, ΔE refers to the resistance, capacitance, and voltage change ΔR, ΔC, ΔU, and $E_{0}$ refer to the initial value of each electrical quantity.

Table 2 provides a summary of representative NW-based pressure sensors in recent years, which are based on different sensing mechanisms and types of materials. Meanwhile, their essential parameters and features are compared.

#### 2.2.1. Resistive Pressure Sensors

Resistive pressure sensors have a simple structure and good linearity. In recent years, structural design has been widely used to improve resistive pressure sensors’ sensitivity, range, and resolution [50,94,95,96,97].

**Figure 3:**
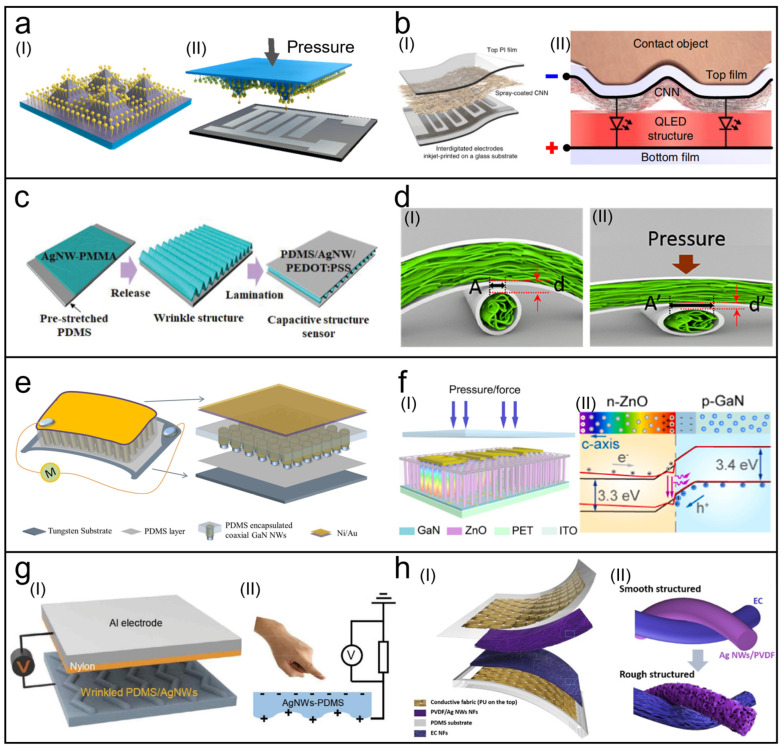
NW-based wearable pressure sensors. (**a**,**b**) Resistive pressure sensors. (**a**) Schematic illustrations of the vertical AuNWs layer growing on micropatterned PDMS (**I**) and the structure of the resistive pressure sensor (**II**). Reprinted with permission from [94]. Copyright 2019, American Chemical Society. (**b**) (**I**) Exploded schematic of CNN-based pressure sensors. (**II**) Working principle schematic of the electroluminescent skin for pressure distribution imaging. Reprinted with permission from [50]. Copyright 2020, Springer Nature. (**c**,**d**) Capacitive pressure sensors. (**c**) Schematic illustration of fabricating processes for the capacitive pressure sensor with a wrinkled dielectric layer. Reprinted with permission from [98]. Copyright 2019, Wiley-VCH. (**d**) Mechanism schematic of AgNW-bacterial cellulose composite fiber-based capacitive pressure sensor. (**I**) At pressure-free state, two fibers are in touch within a small overlap area. (**II**) When pressure is applied, the overlap area significantly increases. Reprinted with permission from [99]. Copyright 2020, American Chemical Society. (**e**,**f**) Piezoelectric pressure sensors. (**e**) Schematic drawing of flexible self-powered piezoelectric pressure sensors based on radial p-n junction GaN NWs. Reprinted with permission from [100]. Copyright 2021, Elsevier B.V. (**f**) Schematic illustrations of p-GaN film/n-ZnO NW LED-based pressure sensor array for pressure distribution mapping (**I**), and energy band of p-GaN/n-ZnO heterostructure after applied compressive strain (**II**). Reprinted with permission from [53]. Copyright 2019, Elsevier Ltd. (**g**,**h**) triboelectric pressure sensors. (**g**) (**I**)Schematic illustration of the conducting-wrinkle-based pressure sensor. (**II**) Circuit diagram of the pressure sensor Reprinted with permission from [101]. Copyright 2019, Wiley-VCH. (**h**) (**I**)Exploded schematic of pressure sensor textile. (**II**) Schematic illustrations of the comparison of the triboelectric nanofibers with the smooth and rough surface. Adapted with permission from [102]. Copyright 2020, American Chemical Society.

Sensitivity is a key indicator of pressure sensors, especially for small-scale pressure applications like pulse detection. In wearable applications, pressure sensors require a wide range and high sensitivity due to the multiscale characteristics of pressure. Microstructures have been proven to enhance the sensitivity and range of pressure sensors. Figure 3a showcases a pressure sensor with a vertical AuNW layer grown on a pyramidal microstructure array using the bottom-up self-assembly growth strategy [94]. This design exhibits a high sensitivity of $23{\;\mathrm{kPa}}^{-1}$ at low pressures (<600 Pa) and maintains a sensitivity of $0.7\;{\mathrm{kPa}}^{-1}$ at higher pressures (600–3000 Pa). Additionally, it demonstrates a fast response time of less than 10 ms during the loading and unloading processes. To further enhance the stability of the sensor, Zhu et al. adopted a spinosum microstructure to improve sensitivity and utilized rGO-wrapped CuNWs as the sensitive material [97] because CuNWs are prone to oxidation in the surrounding environment. The sensor achieved stability in wearable scenarios by employing a simple solution-based method to wrap CuNWs with rGO.

A high spatial resolution is crucial for large-area wearable pressure sensors, but achieving high-pixel-count sensors remains challenging due to material and fabrication limitations. Recent research has employed pixel-less designs in wearable pressure sensors to achieve a high spatial resolution [50,96]. Figure 3b presents a high-resolution pressure sensor comprising a cellulose/NW nanohybrid network (CNN) film and a quantum dot-based electroluminescent film [50]. Te-PEDOT:PSS NWs are utilized in the CNN film. Under applied pressure, the CNN film exhibits a linearly proportional conductivity distribution corresponding to the pressure distribution. This high-resolution pressure sensor enables rapid (<1 ms) and super-resolution (>1000 dpi) electroluminescent imaging under bias voltage.

#### 2.2.2. Capacitive Pressure Sensors

Capacitive pressure sensors are devices that measure the change in capacitance caused by the deformation of the electrodes and the dielectric layer under pressure. Structural and material design for capacitive wearable pressure sensors for electrodes and dielectric can help improve sensitivity [98,103,104,105,106,107].

As shown in Figure 3c [98], by attaching an AgNWs/PMMA film to a pre-stretched PDMS film, wrinkles are formed on the AgNWs/PMMA film when the pre-stretched PDMS is released. Then, an additional layer of the AgNWs–PEDOT:PSS/PDMS film is attached as the top electrode, creating a capacitive pressure sensor with a wrinkled dielectric layer. This design achieves a high sensitivity of up to $2.76\;{\mathrm{kPa}}^{-1}$ at low pressures (<100 Pa), because the wrinkles can increase the contact area and the effective dielectric constant of the sensor.

Furthermore, dielectric design has also explored porous structures to enhance sensitivity [103,106]. Wang et al. prepared a dissolvable porous AgNWs/PVA hydrogel-based capacitive sensor using a facile foaming and freeze-thawing combined method [106], which exhibits a high sensitivity of up to $1.9\;{\mathrm{kPa}}^{-1}$ and a rapid response time of 25 ms, while being capable of dissolving in hot water within minutes. By doping ZnO NWs into the dielectric, the low-pressure sensitivity of the capacitive pressure sensor can be significantly improved (≈6.6 times) thanks to the increased effective dielectric constant and piezoelectric properties of the hybrid dielectric [107].

Textile-based capacitive sensors can be realized by the mutual crossing of conductive yarns. Figure 3d demonstrates a porous AgNWs/bacterial cellulose fiber-based capacitive pressure sensor [99]. The fiber is prepared by continuous wet spinning and achieves a linear high sensitivity response of $5.49\;{\mathrm{kPa}}^{-1}$ in a low-pressure state (<0.5 kPa). The porous structure of the fiber can provide more air gaps for deformation and increase the capacitance change under pressure. Textile-based capacitive sensors have the advantages of flexibility, breathability, and comfortability, making them suitable for wearable applications.

**Table 2:** Summary of NW-based wearable pressure sensors with their parameters and features.

Authors	Sensing Mechanism	Materials	Maximum Sensitivity	Response Time	Durability	Features
Zhu et al. [94]	Resistive	AuNWs/PDMS	23 ${\mathrm{kPa}}^{-1}$ (600 Pa)	<10 ms	>10,000	High sensitivity in a low-pressure regime
Lee et al. [50]	Resistive	Te-PEDOT:PSS NWs/PI	>5000 ${\mathrm{kPa}}^{-1}$	<1 ms	>14,000	High spatial resolution, Transparent
Chen et al. [98]	Capacitive	AgNWs/PDMS	2.76 ${\mathrm{kPa}}^{-1}$	<150 ms	>1000	Low detection limit
Guan et al. [99]	Capacitive	AgNWs/PDMS	5.49 ${\mathrm{kPa}}^{-1}$	<75 ms	>2000	Fibrous sensor, Proximity sensing
Waseem et al. [100]	Piezoelectric	GaNNWs/PDMS	14.25 V/kPa	<55 ms	>250,000	Capability to detect static pressure, Self-powered
Kang et al. [101]	Triboelectric	AgNWs/PDMS	1.187 V/kPa	<30 ms	>900	Self-powered

#### 2.2.3. Piezoelectric/Piezo-Phototronic Pressure Sensors

Due to the high sensitivity of piezoelectric sensors to dynamic stimuli and their self-powered properties, some NWs materials with piezoelectric properties such as ZnO, PZT, MoS_2_, and PVDF have been used as wearable piezoelectric pressure sensors [108,109,110].

It is difficult for piezoelectric pressure sensors to measure static pressure due to the internal shielding of the piezoelectric charge in the piezoelectric material, leading to the attenuation of piezoelectric polarization [111]. To address this internal shielding issue, Figure 3e presents a pressure sensor for static pressure measurements based on GaN/p-GaN coaxial NWs [100]. By growing an Mg-doped p-GaN shell on GaN NWs, a high-quality p-n homojunction and high electrical resistance of p-GaN are achieved. The fabricated sensor can measure static pressure signals over a long duration (>400 s).

High-resolution wearable pressure sensors pose a challenging task. Pressure sensors fabricated based on NW arrays with nanoscale cross-sectional dimensions can achieve extremely high sensitivity. Figure 3f presents a high-resolution wearable pressure sensor based on the piezo-phototronic effect [53]. The piezo-phototronic effect is a phenomenon that uses the piezoelectric potential generated by applying a strain to a semiconductor to control the carrier generation and recombination at the junctions, thus improving the optoelectronic device’s performance. The pressure sensor is made of a flexible p-GaN film/n-ZnO NW heterostructure. When pressure is applied, a positive piezoelectric polarization charge is generated at the p-n junction, changing the energy band structure and promoting the recombination of electrons and holes (Figure 3(fII)). By detecting the luminescence intensity, the pressure distribution can be mapped. The sensor has high sensitivity and a high response speed, making it suitable for wearable electronic devices.

#### 2.2.4. Triboelectric Pressure Sensors

The triboelectric effect is a type of contact electrification, where two materials become electrically charged after being separated from physical contact. During contact electrification, charge moves by electron transfer, the direction of which depends on the materials involved. Based on the triboelectric effect, Triboelectric nanogenerators (TENGs) are energy-harvesting devices that can convert mechanical energy into electrical energy [112,113,114]. TENGs are commonly used as wearable energy-harvesting devices that can power various electronic devices by harvesting human motion energy. However, they can also serve as self-powered wearable pressure sensors that can detect and monitor various physical signals, such as touch, force, vibration, and sound. The sensing mechanism is based on the change in triboelectric charge and electric potential induced by the contact and separation of the triboelectric layers under external pressure. By measuring the output voltage or current of the TENGs, pressure information can be obtained. TENGs are commonly used as wearable energy harvesting devices. However, they can also serve as self-powered wearable pressure sensors. The sensing mechanism is based on the triboelectric effect generated by friction [115].

To enhance this effect and improve the sensitivity, an effective method is to introduce patterned microstructures in the triboelectric layer and electrode [101,116]. Figure 3g showcases a hierarchical-wrinkle structure resembling fingerprints [101]. By applying strain to a PDMS film embedded with AgNWs and subjecting it to Ar plasma treatment, controllable periodic wrinkles with a herringbone pattern are achieved to enhance the triboelectric effect. Figure 3h presents a friction-based textile sensor where an EC nanofibrous membrane (EC-NFM) and a PVDF/AgNWs nanofibrous membrane (PVDF/AgNWs NFM) serve as the positive and negative triboelectric layers [102], respectively. By roughening the textile fibers, a high sensitivity of $1.67\;V\cdot {\mathrm{kPa}}^{-1}$ is achieved within a low-pressure range of 0–3 kPa.

### 2.3. Challenges and Future Opportunities

Based on advancements in fabrication processes and assembly methods, significant progress has been made in NW-based mechanical sensors. These sensors can be classified into resistive, capacitive, piezoelectric, and triboelectric types depending on the sensing mechanism employed. Resistive and capacitive sensors are suitable for monitoring static and low-frequency stimuli, with resistive sensors typically offering higher sensitivity and capacitive sensors exhibiting higher stretchability. Piezoelectric and friction-based sensors, on the other hand, are suitable for monitoring dynamic and high-frequency stimuli and can also achieve self-powering capabilities. By employing design strategies such as microstructures, wrinkling, cracks, and air gaps, NW-based mechanical sensors can further enhance their performance in sensing small-scale stimuli. Moreover, the use of core-shell structures can improve the ability of piezoelectric mechanical sensors to sense static stimuli.

## 3. NW-Based Wearable Photodetectors

Photodetectors have a wide range of applications in wearable electronics, including health detection, image sensing, machine vision, and optical communications. Furthermore, applying photodetectors onto flexible surfaces creates systems that mimic artificial skin, which can enable wearable applications that can achieve high-precision health detection without affecting people’s daily activities [56,117,118]. In addition, wearable image sensors are also widely applied in machine vision and optical communication [119,120,121,122]. NWs exhibit excellent optoelectronic properties due to their large surface-to-volume ratio and high-quality crystal structure, which enhance the transport of charge carriers. Furthermore, with the additional advantages of excellent device strength and the ability to form heterostructures, they are applicable materials for developing photodetectors with high responsivity and specific detectivity. This section introduces the recent developments in the field of NW-based wearable photodetectors, which are divided into photoconductors and photodiodes based on different architectures and working mechanisms. Representative works of NW-based wearable photodetectors and comparisons of their parameters are demonstrated in Table 3.

### 3.1. Photoconductors

**Figure 4:**
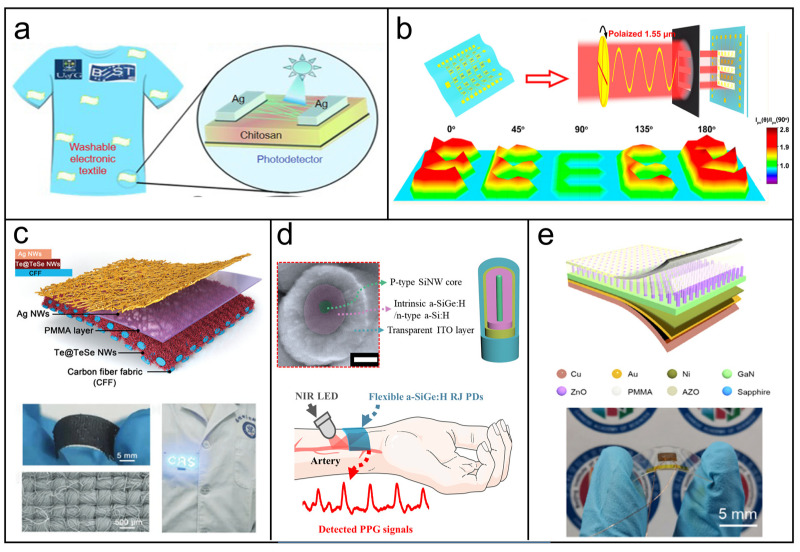
NW-based wearable photodetectors. (**a**,**b**) Photoconductors. (**a**) Schematic illustration of biodegradable and disposable UV photodetectors for smart textiles. Reprinted with permission from [118]. Copyright 2023, Institute of Optics and Electronics, Chinese Academy of Sciences. (**b**) Schematic illustration of the flexible image sensor under polarized 1.55 μm light. Reprinted with permission from [122]. Copyright 2022, American Chemical Society. (**c**–**e**) photodiodes. (**c**) Schematic illustration of the wearable Te@TeSe photodetector textile. Reprinted with permission from [121]. Copyright 2021, Wiley-VCH. (**d**) Schematic illustration of the a-SiGe:H radial junction flexible photodetector and its detecting pulse at the wrist. Reprinted with permission from [56]. Copyright 2022, Wiley-VCH. (**e**) Schematic illustration and an optical image of the flexible UV photodetectors based on piezo-phototronic effect-enhanced photoresponse. Reprinted with permission from [124]. Copyright 2022, Elsevier Ltd.

The mechanism of photoconductors is based on the semiconductor photoconductive effect, which means the conductivity increases by generating free charge carriers induced by light within the semiconductor. In this process, the electric field is applied directly to the active materials and causes the movement of charge carriers [123]. ZnO NWs in II–VI compounds have a wide bandgap of 3.37 eV and a high carrier mobility, resulting in their frequent usage in UV photodetectors. As shown in Figure 4a, Yalagala et al. [118] presented a high-performance UV photodetector based on ZnO NWs, which can serve as a wearable patch to monitor exposure to UV radiations. The device exhibited an excellent photo response, including high responsivity (55 A/W), excellent specific detectivity ($4\times 10^{14}$ jones), and high gain ($8.5\times 10^{10}$). Additionally, it possessed degradability, which is derived from the flexible chitosan substrate.

As another familiar series of materials, III-Sb NWs have a narrow bandgap and very high carrier mobility, which give rise to their utilization for infrared detection. High-quality GaSb NWs with ultrahigh hole mobility can be grown by employing surfactant-assisted chemical vapor deposition [125,126]. Based on the polarized photoresponse properties resulting from the anisotropic 1D geometry structure, Ren et al. [127] presented the application of ordered GaSb nNW arrays in near-infrared polarization photodetection. Furthermore, in order to solve the problem of the excessive dark current of GaSb NW-based photodetectors, a photodetector based on S-GaSb is demonstrated in Figure 4b. This device has excellent polarization imaging capability for incident polarization of 1.55 μm light [122].

**Table 3:** Summary of NW-based wearable photodetectors and their parameters.

Authors	Sensing Materials	Wavelength (nm)	$\mathrm{Responsivity}\;({\mathrm{AW}}^{-1}$)	Specific Detectivity (Jones)	Rise Time/Fall Time (ms)	External Quantum Efficiency (%)
Yalagala et al. [118]	ZnO NWs	365	55	$4\times 10^{14}$	700/800	$10^{4}$
Zhang et al. [122]	S-GaSb NWs	1550	939	$1.1\times 10^{11}$	50	−
Ren et al. [127]	GaSb NWs	1550	77.3	$1.14\times 10^{10}$	−	$0.618\times 10^{4}$
Li et al. [121]	Te@TeSe NWs	1550	−	−	2500/2000	−
Zhang et al. [56]	a-SiGe:H NWs	800	0.14	−	3.6/13.2	−
Peng et al. [124]	GaN/ZnO NWs	325	2.82	$6.82\times 10^{13}$	6.9/6.4	−

### 3.2. Photodiodes

Photodiodes are mainly based on the photovoltaic effect. When light shines on the junction of two different materials, photon energy is absorbed by electrons to generate carriers, such as electron–hole pairs. The photogenerated carriers will generate photocurrent under the influence of the built-in potential or bias voltage across the junction. Photodiodes have the advantages of high operating speed and a linear response to light intensity. Moreover, the heterostructure can significantly improve the photocurrent and responsivity of the photodetector by creating a larger depletion region and a lower barrier for carrier transport. As demonstrated in Figure 4c, Li et al. [121] grew a Te@TeSe NW array on carbon fiber fabric and fabricated photodetectors, which avoided surface damage to fiber photodetectors during the braiding process. Compared with the original TeNW array, the Te@TeSe photodetector fabric enhanced the photocurrent and responsivity by 400 times at 1550 nm.

Vertical NW arrays enable enhanced light absorption performance owing to the amplified light-trapping effect, which is advantageous for the development of high-performance photoelectric devices. Recently, Zhang et al. [56] proposed a 3D hydrogenated amorphous silicon germanium (a-SiGe:H) radial junction (RJ) photodetector constructed over vertical Si NWs on soft Al foils, which can be used as a PPG (photoplethysmography) biosensor under near-infrared light (~800 nm) (Figure 4d). The photodetector can be snugly attached to the human wrist surface for pulse PPG pulse signal detection based on arterial blood volume vibration. Due to the very thin absorber a-SiGe:H layer within the radial junction structures, the photogenerated carriers within the radial p-i-n junction can be effectively and rapidly separated, resulting in a fast response of 3.6 μs/13.2 μs.

The piezo-photoelectric effect can be used to modulate the generation, separation, transportation, and recombination of carriers at the interface or junctions, thus enhancing the performance of NW-based PDs [128,129]. By combining the laser lift-off of the GaN thin film and the hydrothermal synthesis of ZnO NWs, Peng et al. [124] demonstrated a self-powered flexible UV PD based on the p-GaN/n-ZnO heterostructure, which exhibited an improved photocurrent performance with the increase in compressive strain (Figure 4e). Under UV illumination of 38.4 mW/cm^2^, the PD achieved a relative responsivity enhancement of approximately 22% with a compressive strain of −0.48%, which was attributed to the piezo-photoelectric effect. The piezo potential generated by the strain-induced polarization charges in ZnO NWs could modulate the Schottky barrier height at the p-GaN/n-ZnO interface, thus facilitating the separation and transportation of photogenerated carriers.

### 3.3. Challenges and Future Opportunities

In this chapter, we review the development of NW-based photodetectors from two different mechanisms: photoconductors and photodiodes. On the one hand, we study the latest applications of different semiconductor materials on photodetectors from the near-infrared to ultraviolet light range. On the other hand, we investigate the unique mechanical properties, highly ordered integrated arrays, and the ability to form heterostructures of NWs. At the end of this chapter, it is mentioned that the strategy of using the piezo-photoelectric effect to improve the photocurrent and photoresponsivity of FPD belongs to the content of strain engineering. In addition, electrical-field modulation engineering and optical manipulation engineering are also effective strategies to enhance PD performance [130]. In summary, based on the excellent mechanical and optoelectronic properties of 1D structures and many strategies such as strain engineering, NWs will be more widely used in the field of flexible photodetectors. However, there are still some challenges in future practical applications. For example, the large-scale preparation of ordered NW arrays with controllable diameters and shapes to meet the needs of integration and to balance the overall performance of the device and the preparation cost should be paid more attention in future research.

## 4. NW-Based Wearable Temperature Sensors

Temperature is an important and common physical signal in our daily lives. Hence, temperature sensors are being researched in various fields. In terms of wearable applications, wearable temperature sensors are developed rapidly because the accurate detection of body temperature is a significant part of human health monitoring. At present, NWs with excellent thermal properties have been used as a kind of sensing material for temperature sensing [131]. In this section, a series of typical NW-based wearable temperature sensors with different mechanisms and materials are introduced briefly. Table 4 provides a summary of these sensors, and part of their essential parameters are compared.

### 4.1. Thermoresistive Temperature Sensors

**Figure 5:**
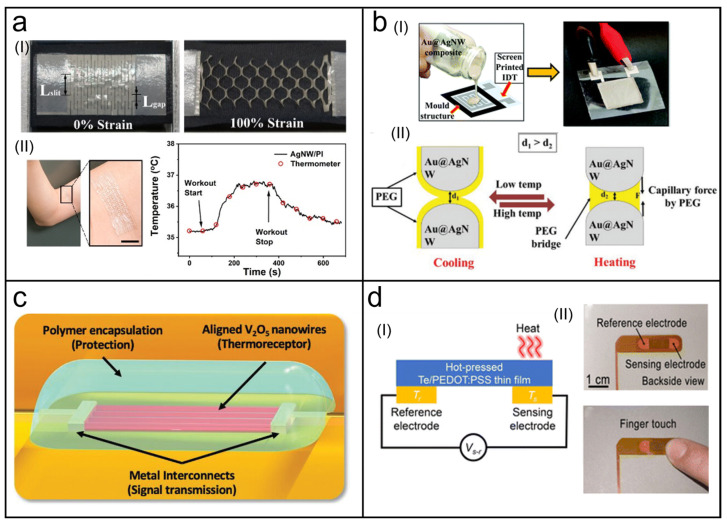
NW-based wearable temperature sensors. (**a**) (**I**) Optical images of a temperature sensor based on AgNWs and PI at tensile strains of 0 and 100%, respectively. (**II**) Optical images of the as-established sensor attached to the skin of a male’s bicep (left). Scale bar: 10 mm. Temperature data were recorded by the sensor and an infrared thermometer during the workout on the biceps (right). Reprinted with permission from [133]. Copyright 2019, American Chemical Society. (**b**) (**I**) Schematic of a temperature sensor fabricated by printing Au@AgNW–PEG–PU nanocomposite ink on interdigitated electrodes. (**II**) Temperature-sensing principle diagram of the fabricated sensor. Reprinted with permission from [55]. Copyright 2022, The Royal Society of Chemistry. (**c**) A schematic illustration of artificial thermoreceptors (ATRs) based on aligned V_2_O_5_ NWs. Reprinted with permission from [134]. Copyright 2022, Wiley-VCH. (**d**) (**I**) Schematic of the sensing principle based on the thermoelectric effect of a hot-pressed Te/PEDOT:PSS composite thin film. (**II**) Photograph of the developed temperature sensor, which has the capability to detect finger temperature. Reprinted with permission from [135]. Copyright 2023, The Royal Society of Chemistry.

The most common mechanism of temperature sensing is the thermoresistive effect, that is, the resistance of the sensor varies with the temperature. Due to the high thermal conductivity of silver, many researchers have been attracted to wearable temperature sensors based on AgNWs. For instance, Jo et al. fabricated a hybrid film using supersonically sprayed AgNWs and electrospun metal-plated (Cu or Ni) nanofibers to act as a temperature sensor [132]. The overall resistance of the film changed obviously with the increase in temperature. Benefiting from the flexibility of this ultra-thin hybrid film, its application as a wearable skin temperature sensor was demonstrated with good performance. In addition to flexibility, another factor that must be considered when designing wearable sensors is stretchability. Cui et al. developed a stretchable thermoresistive temperature sensor based on AgNW networks [133]. As shown in Figure 5(aI), an AgNW percolation network was encased in a thin polyimide film, followed by being cut into kirigami patterns, which granted large stretchability to the film (up to 100%). As external tensile strain was applied to the sensor, the kirigami patterns effectively released the local strain applied on the AgNW network, resulting in outstanding resistance stability under tensile, which is why the sensor could achieve accurate temperature sensing even if it was highly stretched. Figure 5(aII) presents the capability of this temperature sensor in wearable applications. The sensor was attached to the skin of a male’s bicep, which was severely deformed during the workout. However, it could still maintain normal measurements, and the results were basically the same as those obtained by commercial thermometers.

Temperature sensors that incorporate NWs with other substances (polymers, etc.) to enhance sensing performance are also familiar. As shown in Figure 5(bI), a temperature sensor was fabricated by printing Au@AgNW–poly–ethylene glycol (PEG)–polyurethane (PU)-based nanocomposite ink on interdigitated electrodes [55]. Among them, Au@AgNWs and PEG played roles of temperature sensing together, and the sensing principle is shown in Figure 5(bII). Different from the two temperature sensors mentioned above, NWs in this sensor were not connected tightly to each other. As the temperature increased, PEG melted and produced capillary forces between adjacent AgNWs, which reduced the inter-NW distance, resulting in a decrease in resistance. In contrast, PEG crystallized, causing the resistance to increase as the temperature was reduced. Due to the special sensing mechanism brought by PEG, the sensitivity of this sensor was much higher than that of the Au@AgNW film alone. As a wearable temperature sensor, sensing tests on the hand and forehead of a volunteer demonstrated its sensing capabilities comparable to commercial infrared temperature guns. As presented above, AgNW-based temperature sensors exhibit excellent temperature sensing abilities.

Another popular material is metal oxide semiconductors because of their high sensitivities and excellent stable temperature sensing characteristics. However, it is challenging to assemble NWs based on these materials at well-defined locations with good reproducibility and uniformity [136]. Taking this problem into account, Neto et al. reported an e-skin system composed of V_2_O_5_ NW-based artificial thermoreceptors (ATRs) [134] (Figure 5c). A high-density aligned assembly of V_2_O_5_ NWs at specified positions was realized by a dielectrophoresis technique to significantly increase device-to-device uniformity and sensitivity in temperature sensing performance. Relying on the thermoresistive effect, the ATRs could measure the temperature of human skin accurately. Further, the response to temperature stimuli was realized on a robotic hand by utilizing output signals of the ATRs together with learning at a hardware level.

### 4.2. Thermoelectric Temperature Sensors

Another notable temperature-sensing mechanism is the thermoelectric effect. Sensors based on this mechanism can convert ambient temperature gradients into output voltages, thus realizing temperature sensing. Thermoelectric materials are required to be the sensitive materials of thermoelectric temperature sensors. As a series of semiconductor materials with a high Seebeck coefficient, tellurium and a portion of its compounds are well-qualified. Zeng et al. fabricated a high-performance temperature sensor based on interlocking silver telluride (Ag_2_Te) NW network films supported by a nylon substrate, which was capable of recording temperature changes in real-time as a human finger was on-contact/off-contact with the sensor [137].

Similarly, Li et al. replaced the sensitive material with a hot-pressed Te/PEDOT:PSS thin film to measure finger temperature using the same principle [135] (Figure 5d). The developed sensor clarified the reference end and the sensing end, allowing for more accurate temperature identification. Research on thermoelectric temperature sensors is of magnificent significance on account of their ability to recycle waste heat and convert it into valuable electricity. They are able to serve as self-powered temperature sensors or thermoelectric generators [138], both having valuable application prospects in self-powered wearable electronics.

### 4.3. Challenges and Future Opportunities

Compared with continuous films, NW network films have better flexibility and stretchability while maintaining excellent thermal properties such as high sensitivity and quick responses with lower densities. However, there is still room for further improvement in the aspects of resolution, durability, repeatability, and accuracy, which is also a problem for all types of temperature sensors [36]. In particular, for wearable sensors, issues such as biocompatibility and comfortability also require additional consideration. The NW-based temperature sensors are mainly in accordance with two sensing mechanisms, namely the thermoresistive effect and thermoelectric effect, respectively. Sensors based on the former have been widely used because of their simplicity. However, this type of temperature sensor can be easily interfered with by external strains for wearable applications. Reasonable methods (such as structural design) are needed to create strain-insensitive characteristics. The sensors based on the thermoelectric effect take voltage as the output signal, which effectively avoids the problem of crosstalk between temperature and strain. In addition, they have the potential for self-powered wearable electronics as long as the energy can be efficiently harvested.

**Table 4:** Summary of NW-based wearable temperature sensors and their essential parameters.

Authors	Sensing Materials	Structure Type	Sensitivity	Response Time
Jo et al. [132]	AgNWs, metal-plated (Cu or Ni) nanofibers	Network	$0.0783\;{℃}^{-1}$	−
Cui et al. [133]	AgNWs	Network	$0.47\;\Omega \;{℃}^{-1}$	−
Kumar et al. [55]	Au@AgNWs, PEG	Network	~− $0.05\;{℃}^{-1}$	<100 s
Neto et al. [134]	V_2_O_5_ NWs	Planar arrays	− $1.1\;\pm \;0.3\%\;{℃}^{-1}$	≈1 s
Zeng et al. [137]	Ag_2_Te NWs	Network	− $129.5\;{\mu V\;K}^{-1}$	1.05 s
Li et al. [135]	TeNWs, PEDOT:PSS	Network	$302\;\pm \;1\;{\mu V\;K}^{-1}$	1.8 s

## 5. Other Types of NW-Based Wearable Physical Sensors

**Figure 6:**
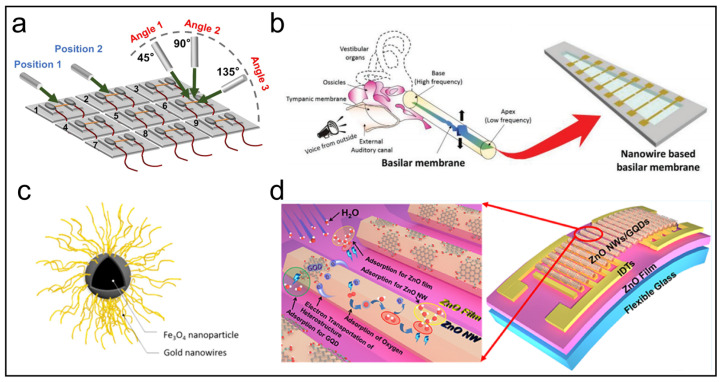
(**a**) Airflow sensor array based on single cm-SiNW. Reprinted with permission from [139]. Copyright 2021, American Chemical Society. (**b**) Resistive acoustic sensor with point crack on PDMS embedded with V-AuNWs. Reprinted with permission from [57]. Copyright 2020, Wiley-VCH. (**c**) Schematic illustration of a Fe_3_O_4_@AuNWs nanoparticle, which provides additional magnetic sensing functionality. Reprinted with permission from [140]. Copyright 2019, Elsevier B.V. (**d**) Diagrams that reveal the structure and sensing mechanism of a SAW humidity sensor based on ZnO NWs and GQDs. Reprinted with permission from [59]. Copyright 2020, American Chemical Society.

Perceiving airflow is an important function of human hair. Figure 6a presents a highly sensitive and imperceptible airflow sensor [139]. The sensitive element of this sensor is composed of cm-SiNWs prepared through the temperature-gradient-assisted VLS method. Due to its extremely high aspect ratio, cm-SiNWs undergo significant deformation in the airflow. The excellent piezoresistive characteristics of SiNWs enable them to generate considerable electrical signals during deformation. By integrating nine sensor units into an array, comprehensive information about airflow, including speed, incident position, and incident angle, can be perceived.

Hearing plays a crucial role in human perception of the external environment. In order to achieve this goal, researchers have developed various acoustic sensors based on NWs, including resistive [57], capacitive [141], triboelectric [142], and piezoelectric [143] NW-based acoustic sensors. Figure 6b showcases a resistive artificial basilar membrane based on the point-cracking method [57]. By introducing point cracks in a vertical AuNW film, this membrane overcomes the frequency discrimination limitations of resistive acoustic sensors and achieves ultrahigh acoustic sensitivity (0.48–4.26 Pa^−1^).

As a novel application, the magnetic field sensing function has recently been involved in several soft sensors. Under normal circumstances, the sensing principle is that the magnetic field causes the resistance to change in soft sensors due to the deformation caused by the movement of magnetic materials inside. Based on this principle, the combination of conductive NWs and magnetic nanoparticles can usually achieve fantastic performance [144,145]. For instance, Yap et al. reported a flexible sensor using Fe_3_O_4_ nanoparticles on which AuNWs were grown [140] (Fe_3_O_4_@AuNWs), as shown in Figure 6c. In addition to the classical pressure and strain sensing, Fe_3_O_4_@AuNWs ink could be printed in a serpentine structure to serve as a magnetic sensor. As this sensor was in a magnetic field, it bent due to the generation of the magnetic force, which led to resistance change, and the degree of the change was related to the strength of the magnetic field. As a wearable application, the magnetic sensor is especially useful for patients who have metal implants such as pacemakers or joint replacements to avoid magnetic fields.

Compared with magnetic field sensing, magnetic nanowires (MNWs), which are magnetic themselves, are more commonly used in the field of cellular biosensing [146,147]. In such applications, MNWs usually serve as biolabels to label cells, and the detection of cells is achieved by sensing magnetic signals on MNWs. For example, Kouhpanji et al. proposed a methodology for the detection of cancer cells adopting NiNWs as biolabels [147]. Specifically, characterized MNWs were surface-functionalized and internalized into canine OSCA-8 cancer cells to accomplish biosensing by measuring irreversible switching fields (ISFs) of NiNWs with a projection method. It is highlighted that MNWs with different ISFs were synthesized in this work, leading to selective measurements. Since only magnetic signatures are detected, the biosensing approaches based on MNWs enable the signals to be less contaminated by background noises, resulting in an excellent signal-to-noise ratio. In addition to biosensing, MNWs are also well-qualified in fields involving cell manipulation, cell separation, drug delivery, and cancer therapy, having broad prospects for future developments [147,148].

Humidity sensors also have a wide range of applications since humidity is an important notable physical signal in aspects of human lives, such as detecting breathing rate and fingertip moisture. Recently, a large number of high-performance humidity sensors based on a variety of sensing mechanisms, such as resistive [149], capacitive [150], and surface acoustic wave (SAW) [59] sensors, have been reported using various materials. NWs are materials with a large specific surface area, which provide remarkable humidity-sensing capabilities by absorbing a relatively large number of water molecules. As a typical example of SAW humidity sensors, a hybrid sensitive film based on ZnO NWs and graphene quantum dots (GQDs) is proposed (Figure 6d) [59]. The water absorption of the sensitive film is the key to humidity sensing because it is the mass load on the surface of the film that affects the transmission characteristics of SAW devices. Therefore, in addition to ZnO NWs, GQDs are also used to provide superior water absorption due to their large number of hydrophilic functional groups and the formation of p-n heterojunctions between GQDs and ZnO NWs. Wearable applications of this sensor achieved high sensitivity, stability, and reliability, which revealed potential applications for human–machine interaction, spatial localization, and personal healthcare.

## 6. NW-Based Multifunctional Wearable Physical Sensors

As the demand for detecting multiple signals from the human body increases, sensors that only respond to a single signal are no longer sufficient to meet the new challenges of wearable electronics, such as miniaturization, integration, intelligence, etc. [151]. Therefore, many pioneering efforts have been devoted to multifunctional wearable sensors with high sensitivity and accuracy in recent years. In this section, we present some outstanding recent works on NW-based multifunctional wearable sensors, which can be classified into two categories: multifunctional single sensors and multifunctional integrated sensors.

### 6.1. Multifunctional Single Wearable Physical Sensors

Multifunctional single wearable physical sensors can detect different types of physical signals with a single sensor, which satisfies the requirement of miniaturization for wearable electronics. This type of multi-functional sensor requires common considerations in material selection and device structure design.

**Figure 7:**
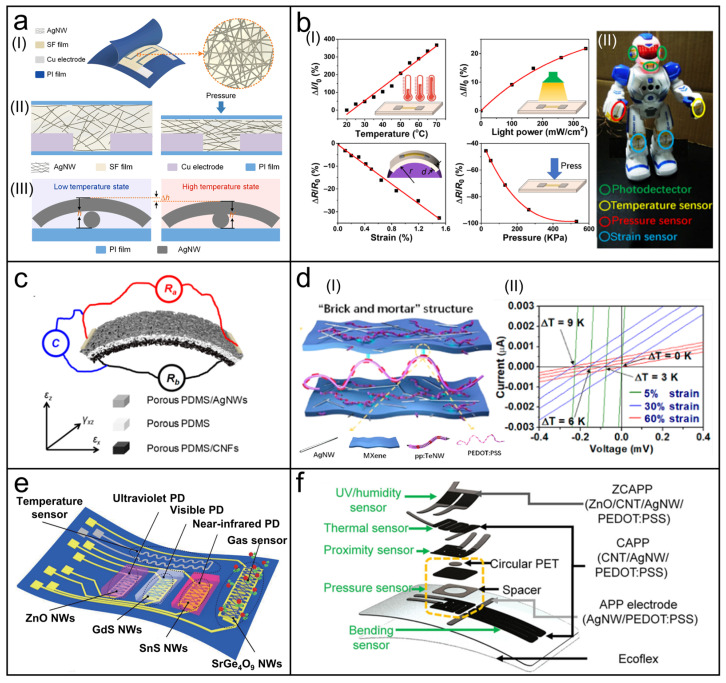
NW-based multifunctional wearable sensors. (**a**) Schematic structure diagram of a multifunctional sensor based on an SF/AgNW composite film (**I**) and schematic structure diagram of its pressure-sensing mechanism (**II**) and temperature-sensing mechanism (**III**). Reprinted with permission from [154]. Copyright 2023, Elsevier Ltd.; (**b**) (**I**) Characterization of the multifunctional sensing properties of the SiNW fabrics. (**II**) Photograph of a robot integrated with three photodetectors, a temperature sensor, a pressure sensor, and two strain sensors, all based on the SiNW fabrics, as a schematic of wearable applications. Reprinted with permission from [155]. Copyright 2019, Springer Nature. (**c**) Conceptual diagram of a multimodal force sensor based on AgNWs, carbon nanofibers, and porous PDMS. Reprinted with permission from [156]. Copyright 2020, American Chemical Society. (**d**) (**I**) Schematic illustration of a stretchable dual-parameter sensor with “Brick and mortar” structure composed of AgNW, Mxene, pp:TeNW, and PEDOT:PSS. (**II**) I–V curves of the multifunctional sensor measured under different ΔT and diverse applied strains. Reprinted with permission from [157]. Copyright 2020, American Chemical Society. (**e**) Schematic diagram of a multifunctional device with a temperature sensor, a gas sensor, and three photodetectors using NWs with diverse materials. Reprinted with permission from [159] Copyright 2020, Springer Nature. (**f**) Exploded schematic of a multilayer multifunctional biosensor based on AgNWs, CNTs, ZnO, and PEDOT:PSS via a supersonic cold-spraying method. Reprinted with permission from [160]. Copyright 2022, Springer Nature.

Some NW materials have been demonstrated to respond to multiple physical signals, allowing the development of corresponding multifunctional wearable physical sensors [152,153]. For instance, Figure 7a presents a flexible multifunctional sensor based on silk fibroin/silver NW (SF/AgNW) composite film [154]. Applying pressure and increasing temperature both decreased the resistance of the film by causing adjacent NWs to come into contact (Figure 7(aI,II)). The developed sensor was, therefore, sensitive to both temperature and pressure. Furthermore, an application of high-quality SiNW fabrics grown in one step using a massive metal-assisted chemical vapor deposition (CVD) method for multifunctional wearable sensors is demonstrated in Figure 7b [155]. A large mass of millimeter-long SiNWs was interwoven, providing flexibility, comfort, and even tailorability. Based on the excellent inherent sensing properties of SiNWs, the SiNW/fabric-based sensor was able to achieve the measurement of four different signals, including temperature, light power, strain, and pressure (Figure 7(bI)). The fabricated sensors could realize different sensing functions according to the application scenarios (Figure 7(bII)), which demonstrated the research significance of this type of multifunctional single sensor.

However, it is difficult to decouple multiple signals in a single sensor, and there might be severe crosstalk between the two sensing modes. Therefore, ingenious structural designs are required to achieve simultaneous measurement of multiple physical signals. As shown in Figure 7c, by depositing AgNWs and carbon nanofibers onto porous PDMS, respectively, a multimodal force sensor based on a sandwich-like structure was developed [156]. The AgNWs and carbon nanofibers formed conductive networks, respectively, so that the upper and lower plates were conductive, which acted as two resistive sensors and two electrodes of a capacitive sensor at the same time. According to three given force–electric relationship expressions, this sensor was able to measure normal pressure, in-plane stretch, and transverse shear forces simultaneously based on the capacitive and two resistive responses. It is also possible to measure unrelated physical signals such as strain and temperature. As shown in Figure 7(dI), a temperature–strain dual-sensing device was reported based on a nanocomposite composed of titanium carbide (MXene), AgNWs, PEDOT:PSS, and TeNWs [157]. The piezoresistive effect of the whole nanocomposite and the thermoelectric effect of TeNWs were the sensing mechanisms of strain sensing and temperature sensing, respectively. Due to different output signals and the insensitivity of the resistance to temperature, simultaneous detection of strain and temperature was successfully achieved by observing the intercept (voltage) and slope (resistance) of the I-V curves of the sensor, respectively (Figure 7(dII)). Based on a similar principle, Li et al. fabricated a bimodal sensor based on vertical TeNW arrays that could decouple pressure and temperature signals [158]. Surprisingly, the pressure-sensing function of the sensor was improved to measure the hardness of a material, while the temperature-sensing function was explored to measure the thermal conductivity. Further, the sensor was used to develop a wearable glove. With three detected signals, including pressure, hardness, and thermal conductivity, it was capable of endowing real objects to the virtual world after using a deep learning technique, which provided new inspiration for the development of virtual reality (VR) technology.

### 6.2. Multifunctional Integrated Wearable Physical Sensors

A simple and feasible approach to detecting multiple signals simultaneously is to integrate several high-performance sensors with capabilities to sense different signals. As shown in Figure 7e, a typical in-plane-integrated NW-based multifunctional sensing platform is presented by Chen et al. [159], which included a temperature sensor based on Ni microwire, a gas sensor based on SrGe_4_O_9_ NWs, and three photodetectors based on ZnO, CdS, and SnS NWs for the detection of ultraviolet, visible, and infrared light, respectively. The crosstalk problem brought by multiple signals still exists but is easy to solve for integrated sensors. In this work, temperature compensation was proposed to address the effect of temperature on other sensors. In addition, light or gas signals were responded to by certain NWs, while other sensors could not be disturbed.

Despite its good performance, the heterogeneous integration of multiple materials can easily lead to damage caused by mechanical mismatches, which brings complexity to the fabrication of integrated sensors. In response to this problem, Gong et al. developed a kind of multifunctional tattoo-like electronic based on vertical AuNW microarray structures [161], which only adopted gold as the sensing material, thereby avoiding the use of complex multimaterial interfaces. The vertical AuNW microarrays were not sensitive to strain due to their specially aligned configuration. However, extremely high strain sensitivity in local areas was achieved by introducing localized cracks, resulting in the feasibility of multifunctional measurements without interference. An integrated pressure sensor, strain sensor, and anisotropic orientation-specific sensor were designed with cracks, while a temperature sensor with a serpentine pattern and a pair of glucose/lactate sensors that utilized a three-electrode system were designed without cracks. With the same consideration, Kim et al. developed a single-material-based integrated system based on two kinds of AuNWs, namely Ag@Au NWs with bulk structures (Ag@AuNWs) and Au hollow NWs with hollow structures (AuHNWs) [162]. The two NWs had better responses to temperature and strain, respectively, due to differences in microstructure. As a result, the Ag@AuNWs were used for the stretchable interconnector, a heater, and a temperature sensor, whereas the AuHNWs were used for a strain sensor. This is an uncomplicated and high-performance application of monolithically patterned NWs in wearable integrated electronics.

Planar integration requires more space to achieve multiple functions, which will limit further miniaturization and higher integration of wearable electronics. Multilayer structures are good candidates for solving this problem. For example, Jo et al. fabricated a multilayer multifunctional sensor based on AgNWs, CNTs, ZnO, and PEDOT:PSS via a supersonic cold-spraying method [160], as presented in Figure 7f. It could detect six signals, including strain, pressure, proximity, temperature, humidity, and UV light, while covering a relatively small area. In addition, adopting multilayer structures alleviated the interference between different signals to a certain extent, resulting in the possibility of measuring multiple signals simultaneously.

### 6.3. Challenges and Future Opportunities

Multifunctional wearable sensors have stricter requirements for sensing materials. NWs are suitable materials because of their diversity and high sensitivity to multiple signals. However, further optimization is important for both NWs and sensing systems. For NWs, the selection of materials, preparation technologies, and assembly process should be carefully considered. For sensing systems, more exploration is needed regarding the design of device structures and integrated circuits. Moreover, new sensing mechanisms are of greater significance for the development of multifunctional sensing. Wearability (e.g., flexibility, stretchability, and breathability) is also worth reasonable design attention as detecting signals on the human body is required.

## 7. Conclusions and Perspectives

In this review, we have summarized the recent representative research works of various types of NW-based wearable physical sensors, including the structural design, sensing mechanisms, manufacture, and practical applications of these sensors, highlighting the positive role that NWs play in the sensing process. We are pleasantly surprised to find that NW-based wearable sensors have a wide range of applications in each category with high performance, which illustrates that their future explorations are worthwhile.

Despite plenty of remarkable progress, challenges remain in this field. For example, achieving flexibility and stretchability while maintaining perfect sensing performance in all aspects is what researchers have been working towards. Moreover, cost is also an issue to consider for efficient and mass production. Here, several future opportunities are presented to serve as possible references for the development of this field:

- Preparation and assembly methods of NWs

Currently, there are relatively mature methods for preparing or growing NWs. However, further improvement is still possible by developing preparation and assembly methods for NWs. The expected outcomes of improvement are either a simple and low-cost approach or higher-quality NWs. High density is an important goal for the assembly of all types of NWs. For NWs formed by self-assembly methods, alignment and controllability are crucial factors. To achieve these goals, some challenges and opportunities need to be addressed. For example, how to control the size, morphology, orientation, component, and position of NWs during the growth process; how to integrate NWs into functional devices or systems with high efficiency and reliability; how to characterize the properties and performance of NWs and their assemblies; and how to explore the novel applications of NWs in various fields.

The resolution and feature size of the patterns are of significant importance for function integration and device performance. There are several approaches to achieving this goal. Lithography techniques enable precise top-down etching of the linewidth of NWs. By employing EBL, high-resolution NW arrays can be achieved. Additionally, NIL could fabricate NW arrays with mass-batching, low cost, and high resolution. In the case of 2D NW networks, achieving high resolution often comes at the expense of damaging the percolation network, leading to reduced conductivity. To address this issue, one approach involves densifying the NW network, while another approach involves aligning the NWs to enhance their conductivity.

- Structural design of NWs and sensors

Reasonable structural design of NWs is able to effectively improve part of the sensing performances. Currently, NWs with various structures such as hollow, hierarchical, porous, kinked, and core-shell NWs have been developed, all demonstrating unique advantages under their respective conditions of use. Hence, more creative NW structures can be designed for special sensing requirements. In addition, the macro-structural design of assembled NW-based sensing films is also conducive to the application in specific cases. Bionic structures are possible to be referenced for both micro and macro design because nature is a wonderful source of inspiration for structural inventions.

- Sensing mechanism

The innovation in sensing mechanisms is particularly challenging. However, a qualitative leap may be introduced for the sensing capability of sensors once breakthroughs arise in this area. For wearable systems, bionic research on animal nervous systems provides a new direction. The analysis of the biosensing mechanism of animal receptors likely lays a new theoretical foundation for the study of high-performance wearable sensors.

- Other issues worth considering

Multifunctional sensing will definitely become a trend in wearable sensor development to satisfy the new requirements of wearable electronics. Nevertheless, crosstalk, as a serious disadvantage, emerges when multiple signals are measured simultaneously. Methods need to be proposed to solve this problem in addition to attaining high sensing performance. For multifunctional single sensors, researchers can try to establish decoupled equations or adopt mutually independent sensing mechanisms and output signals for the detection of different signals. For multifunctional integrated sensors, diverse sensing materials could be applied to detect different signals, which easily avoids interference between individual sensors. Moreover, temperature compensation and similar methods are effective as well.

Artificial intelligence (AI) is a technology that has developed rapidly in recent years. Its ability to learn from data and perform tasks makes it a perfect assistant for wearable sensors. For example, trained AI has the capabilities of quickly integrating and analyzing human data obtained from wearable sensors and providing appropriate conclusions about human health, which is particularly useful in the field of health monitoring and medical diagnosis. In addition, novel functions such as AI motion monitoring and AI voice assistants can also be added to wearable electronics. Wearable sensors assisted by AI will bring great convenience to human lives; therefore, we look forward to the combination of high-performance NWs and AI-assisted wearable sensors in future advances.

In conclusion, NW-based wearable physical sensors are an emerging and exciting research field, which will bring significant scientific and technological progress and social well-being to human society. They provide the potential to revolutionize the fields of healthcare, human–machine interface, IoT, VR/AR, and more. We anticipate more future innovations and breakthroughs in this field, as well as more practical applications and product values.

## Data Availability

Not applicable.
